# HIV Broadly Neutralizing Antibodies Expressed as IgG3 Preserve Neutralization Potency and Show Improved Fc Effector Function

**DOI:** 10.3389/fimmu.2021.733958

**Published:** 2021-09-10

**Authors:** Simone I. Richardson, Frances Ayres, Nelia P. Manamela, Brent Oosthuysen, Zanele Makhado, Bronwen E. Lambson, Lynn Morris, Penny L. Moore

**Affiliations:** ^1^Centre for HIV and STI’s, National Institute for Communicable Diseases, a Division of the National Health Laboratory Service, Johannesburg, South Africa; ^2^Medical Research Council (MRC) Antibody Immunity Research Unit, Faculty of Health Sciences, University of the Witwatersrand, Johannesburg, South Africa; ^3^Centre for the AIDS Programme of Research in South Africa (CAPRISA), University of KwaZulu-Natal, Durban, South Africa

**Keywords:** broadly neutralizing antibodies (bnAbs), Fc effector function, IgG3, phagocytosis, ADCC (antibody dependent cellular cytotoxicity)

## Abstract

The ability of several broadly neutralizing antibodies (bNAbs) to protect against HIV infection is enhanced through Fc receptor binding. Antibody isotype modulates this effect, with IgG3 associated with improved HIV control and vaccine efficacy. We recently showed that an IgG3 variant of bNAb CAP256-VRC26.25 exhibited more potent neutralization and phagocytosis than its IgG1 counterpart. Here, we expanded this analysis to include additional bNAbs targeting all major epitopes. A total of 15 bNAbs were expressed as IgG1 or IgG3, and pairs were assessed for neutralization potency against the multi-subtype global panel of 11 HIV strains. Binding to the neonatal Fc receptor (FcRn) and Fcγ receptors were measured using ELISA and antibody-dependent cellular cytotoxicity (ADCC) and phagocytosis were measured using infectious viruses and global panel Env SOSIP trimers, respectively. IgG3 bNAbs generally showed similar or increased (up to 60 fold) neutralization potency than IgG1 versions, though the effect was virus-specific. This improvement was statistically significant for CAP256-VRC26.25, 35022, PGT135 and CAP255.G3. IgG3 bNAbs also showed significantly improved binding to FcγRIIa which correlated with enhanced phagocytosis of all trimeric Env antigens. Differences in ADCC were epitope-specific, with IgG3 bNAbs to the MPER, CD4 binding site and gp120-gp41 interface showing increased ADCC. We also explored the pH dependence of IgG1 and IgG3 variants for FcRn binding, as this determines the half-life of antibodies. We observed reduced pH dependence, associated with shorter half-lives for IgG3 bNAbs, with κ-light chains. However, IgG3 bNAbs that use λ-light chains showed similar pH dependence to their IgG1 counterparts. This study supports the manipulation of the constant region to improve both the neutralizing and Fc effector activity of bNAbs, and suggests that IgG3 versions of bNAbs may be preferable for passive immunity given their polyfunctionality.

## Introduction

Antibodies mediate pathogen neutralization through the binding of the Fab portion to antigen, and also elicit several effector functions through interaction of the Fc with a variety of receptors. Both neutralization and Fc effector function have been shown to be critical *in vivo*. The importance of neutralization has been confirmed by passive transfer studies, where bNAbs provide sterilizing immunity in animal models (1–3) and robust antiviral activity in chronically infected humans (4, 5). Despite this, combinations of bNAbs with high levels of potency and breadth will be needed to improve on the results of the antibody-mediated protection (AMP) passive immunization trial with VRC01 (6). Fc receptor engagement results in the recruitment of cytotoxic functions which have been shown in HIV infection to restrict the number of transmitted/founder viruses that establish infection, reduce viral load, and drive viral escape (7–10) and are associated with spontaneous HIV control (11) and slowed disease progression (12). Polyfunctional Fc effector function has also been associated with vaccine protection in humans and non-human primates (13–19) and with the development of broadly neutralizing antibodies (bNAbs) during infection (20, 21). Further, Fc receptor binding is required for several bNAbs to optimally protect from infection or clear infected cells in different animal models (22–28). Thus, while the elicitation of HIV bNAbs is likely necessary for an efficacious vaccine, Fc effector function can complement this function to improve efficacy.

Among the factors that contribute to modulating Fc effector function is antibody isotype (IgM, IgA, IgG and IgE) and subclass (IgG1-4 and IgA1-2), determined by sequence variation in the constant regions of the heavy chain (CH1-3) genes. The unique structures of each isotype result in differential binding to multiple Fc receptors, and this translates to diversity of Fc effector functions, varying half-lives and immune complex formation (29). Furthermore, there is substantial evidence that isotype can significantly alter antigen affinity and/or neutralization capacity of monoclonal antibodies (30–35), indicating the importance of the isotype well beyond Fc receptor binding. Isotype therefore potentially represents an important factor to improve the function of bNAbs for passive immunization.

Of the IgG subclasses, IgG3 antibodies are the most polyfunctional, owing to their increased affinity for Fc receptors (36). IgG3 is highly polymorphic with 29 reported alleles (37), and this variability is known to alter antibody activity and half-life (34, 38, 39). Structurally, IgG3 is distinct from other subclasses, with a long flexible hinge, enabling high rotational freedom about the Fc-Fab and Fab-Fab axes (40, 41). In HIV infection, skewing towards IgG3 has been associated with reduced risk of infection in the RV144 and HVTN 505 vaccine trials (14, 15, 42) and in viral control (11). IgG3 specific bNAbs have also been shown to mediate greater antibody-dependent cellular phagocytosis (ADCP) compared to IgG1 (43, 44), largely through their elongated hinge (34, 45). In addition to better ADCP, we previously demonstrated that IgG3 variants of the V2-specific bNAb CAP256-VRC26.25 showed enhanced antibody-dependent cellular trogocytosis (ADCT) and significantly improved neutralization potency when compared to IgG1 (34).

IgG3 is however not currently used for any therapeutic antibodies in a clinical setting. One of the major reasons for this is its reduced half-life of approximately 7 days compared to 21 days for IgG1 (41). Half-life is largely mediated by antibody binding to the neonatal Fc receptor (FcRn). IgG is able to bind FcRn at acidic conditions (pH6) within endosomes. FcRn–IgG complexes are then routed away from the lysosomal degradation pathway, and through the merging of vesicles with the plasma membrane, returned to physiological pH (pH 7.4), where IgG is released (46). Unlike IgG1*01 which contains a histidine at position 435, IgG3 alleles such as IgG3*01 contain an arginine at position 435. This does not deprotonate at neutral pH, resulting in IgG3 binding to FcRn being less pH-dependent (39, 47), an undesirable feature for a therapeutic antibody. However several IgG3 alleles (IgG3*17, *18 and *19), like IgG1, have a histidine at position 435. Furthermore, other variable region structures of the antibody are known to affect half-life (48, 49), demonstrating alternative ways to engineer IgG3 bNAbs with enhanced pH dependence for FcRn binding.

Here, we examine the impact of IgG3 isotype on Fc effector function and neutralization activity of 15 bNAbs that target the five major bNAb epitopes on the HIV trimer. We engineered paired IgG1 and IgG3 variants of the bNAbs and assayed them for ADCP and antibody-dependent cellular cytotoxicity (ADCC) activity. We show that ADCP was globally improved by IgG3, however ADCC was improved in an epitope-dependent manner. We examine the features of IgG3 bNAb light chains that result in similar binding to FcRn compared to their IgG1 counterparts and show that IgG3 has increased binding to Fcγ receptors. Finally we demonstrate that neutralization potency of these IgG3 bNAbs is maintained or enhanced. This suggests that IgG3 variants of bNAbs may be preferable for use in passive immunity as they not only display improved Fc effector function but also show enhanced neutralization potency in a virus-specific manner.

## Materials and Methods

### Ethics

Approval for use of PBMCs from healthy HIV uninfected individuals was approved by the Human Research Ethics Committee of the University of the Witwatersrand (M150313).

### Cell Lines

THP-1 cells obtained from the AIDS Reagent Program (Division of AIDS, NIAID, NIH contributed by Dr. Li Wu and Vineet N. KewalRamani) were used for the ADCP assay. Cells were cultured at 37°C, 5% CO_2_ in RPMI containing 10% heat-inactivated fetal bovine serum (Gibco, Gaithersburg, MD), 1% Penicillin Streptomycin (Gibco, Gaithersburg, MD) and 2-mercaptoethanol to a final concentration of 0.05 mM. CEM-NK_R_.CCR5, a CEM-natural killer resistant T lymphoblast cell line transduced with CCR5 served as targets in the ADCC assay. These were obtained from the AIDS Reagent Program (Division of AIDS, NIAID, NIH developed by Dr Alexander Trkola) and were cultured at 37°C, 5% CO_2_ in RPMI containing 10% heat-inactivated fetal bovine serum (Gibco, Gaithersburg, MD) and 1% Penicillin Streptomycin (Gibco, Gaithersburg, MD). TZM-bl cells, previously designated JC53-bl (clone 13) cells, are a HeLa cell line expressing high levels of CD4 and CCR5 and transduced with a luciferase gene under the control of the HIV promoter. These were obtained from the AIDS Reagent Program (Division of AIDS, NIAID, NIH developed by Dr. John C. Kappes, and Dr. Xiaoyun Wu) and used in neutralization assays. HEK293T cells were obtained from Dr. George Shaw (University of Alabama, Birmingham, AL) and were used for pseudovirus expression. These adherent cell lines were cultured at 37°C, 5% CO_2_, in DMEM containing 10% heat-inactivated fetal bovine serum (Gibco BRL Life Technologies) and supplemented with 50 μg/ml gentamicin (Sigma). Cells were disrupted at confluence with 0.25% trypsin in 1 mM EDTA (Sigma) every 48–72 hours. HEK293F suspension cells were cultured in 293Freestyle media (Gibco BRL Life Technologies) and grown in a shaking incubator at 37°C, 5% CO_2_, 70% humidity at 125rpm.

### Proteins and Peptides

Constructs of avitagged SOSIP trimers of 246.F3.C10.2, BJOX002000.03.2 and CE1176.A3 from the global virus panel (50) were a gift from Dr Christopher Cottrell (The Scripps Research Institute). These were transfected into HEK293F suspension cells with PEIMax, incubated for 6 days in a shaking incubator at 37°C, 5% CO_2_, 70% humidity at 125 rpm and purified by sequential Ni-NTA and size exclusion chromatography (SEC) as described elsewhere (51). Prior to use, trimers were subjected to quality control by ELISA binding of monoclonal antibodies CAP256-VRC26.25 and PGT151 (which trimeric Env forms only) and F105 and 447-D (which do not bind native-like Env trimers). Biotinylated MPR.03 peptide was purchased from Peptide 2.0 (Chantilly, Virginia).

### Antibody Engineering and Production

The heavy and light chain variable regions of bNAbs of interest were cloned into both IgG1 and IgG3 (received from Dr Bart Haynes, Duke University, Durham, NC) expression vectors. The allelic variants of each subclass were IgG1*01 and IgG3*01 respectively. IgG3*01 differs from IgG1*01 with a hinge length of 62 compared to 15 amino acids as well as at many key Fc receptor binding sites. This includes position 435, a key site for FcRn interaction and enhanced half-life for which IgG1*01 contains a histidine and IgG3*01 contains an arginine. For antibody expression, plasmids encoding heavy or light chain genes were co-transfected into HEK293F cells with PEI-MAX 40,000 (Polysciences) head-to-head. Cells were cultured for six days in 293Freestyle media at 37°C, 10% CO_2_, then harvested supernatants were filtered and purified using Protein G (Thermoscientific). Antibody concentrations of all variants were quantified by nanodrop using sequence-specific extinction coefficients as determined by ProtParam (ExPASy) and confirmed by ELISA. SDS-PAGE was used to confirm IgG1 and IgG3 stability and size.

### Antibody-Dependent Cellular Phagocytosis (ADCP) Assay

The THP-1 phagocytosis assay was performed as in (52) using 1 μM neutravidin beads (Molecular Probes Inc, Eugene, OR) coated with 246.F3.C10.2, BJOX002000.03.2 or CE1176.A3 SOSIP trimer or MPR.03 peptide. SOSIP was biotinylated on an avitag to ensure correct orientation when binding to the beads. Antibodies were tested starting at 10 μg/ml with 5-fold dilutions. Phagocytic scores were calculated as the geometric mean fluorescent intensity (MFI) of the beads that have been taken up by THP-1 cells, multiplied by the percentage bead uptake on a FACSAria II (BD Biosciences, Franklin Lakes, New Jersey). Pooled IgG from HIV-positive donors from the NIH AIDS Reagent programme (HIVIG) was used in all assays to normalize for plate to plate variation and Palivizumab (MedImmune, LLC; Gaithersburg, MD) was used as negative control.

### Infectious Antibody-Dependent Cellular Cytotoxicity (ADCC) Assay

The HIV-1 reporter viruses used in the ADCC assays were replication-competent infectious molecular clones (IMC) encoding the 246.F3.C10.2, BJOX002000.03.2 and CE1176 env within an isogenic backbone Env-IMC-6ATRi, that also expresses the Renilla luciferase reporter gene, and preserves all viral open reading frames produced as described previously (53). These constructs were kindly provided by Dr Christina Ochsenbauer (University of Alabama at Birmingham). Reporter virus stocks were generated by transfection of HEK293T cells (NIH AIDS Reagent Program) with proviral IMC plasmid DNA, and titered for infectivity in CEM.NK_R_CCR5 cells (NIH AIDS Reagent Program) by p24 staining (Beckman-Coulter). CD4 downregulation was also measured with co-staining with anti-CD4. ADCC activity as previously described (54). Briefly, a CEM.NK_R_CCR5 cell line (NIH AIDS Reagent Program) was used as the target for ADCC luciferase assays after infection with the HIV-1 IMCs listed above. The target cell line was infected with IMC using titered stocks that generated more than 50% infected cells after 72 hours of infection. These were incubated with 5-fold serially diluted mAbs starting at 50 μg/ml. Cryopreserved peripheral blood mononuclear cells (PBMC) obtained from a HIV-negative donor with a high-affinity 158V/V FcγRIIIa phenotype were used as source of effector cells. After thawing, the cryopreserved PBMCs were rested overnight and used at an effector-to-target ratio of 30:1. The effector cells, target cells, and Ab dilutions were plated in white 96-well half area plates and incubated for 6 hours at 37°C in 5% CO_2_. The final readout was the luminescence intensity (in relative light units) generated by the presence of residual intact target cells that had not been lysed by the effector population in the presence of any ADCC-mediating mAb. The percentage of killing was calculated using the formula:


$$\%\;killing=\frac{\left(RLU\;of\;target\;and\;effector\;well)-(RLU\;of\;sample\;well\right)}{RLU\;of\;target\;and\;effector\;well}\times 100$$

In this analysis, the RLU of the target plus effector wells represents non-antibody background. The RSV-specific mAb Palivizumab (Medimmune; Synagis) and A32 that does not bind to prefusion trimer (NIH AIDS Reagent Program) were used as negative controls and a polyclonal mixture of IgG from HIV infected individuals (HIVIG) from the NIH AIDS Reagent Program was used to normalize between plates. Data are represented as the area under the curve (AUC) of percentage specific killing over the serially diluted antibodies.

### FcγR Binding ELISA

Antibody binding to FcγR was measured by ELISA as described previously (55). Briefly, FcγRI, FcγRIIa, FcγRIIb and FcγRIIIa His6-tagged receptors (R&D Systems Minneapolis, MN) were coated on nickel plates (Qiagen) at 2 μg/ml or 4 μg/ml. Five-fold serial dilutions starting at 5 μg/ml of bNAbs were added. Binding was detected by a goat Anti-Human IgG (Fab specific) antibody goat at 1 in 10,000 (Sigma). Results were visualized with tetramethylbenzidine (TMB).

### Neonatal Fc Receptor (FcRn) ELISA

Binding to the neonatal Fc receptor was measured as described in (24). Nickel plates (Qiagen) were coated with 2 μg/ml his-tagged FcRn/β2 (Sinobiological, Bejing) for a minimum of 1 hour, washed with PBS 0.05% Tween-20, and blocked with 5% milk/PBS. Five-fold serial dilutions starting at 5 μg/ml of bNAbs were incubated with the receptor in 100mM NaPO4, 0.05% (v/v) Tween20, pH 6.0 for 1 hour at room temperature. Following this, plates were either washed with 100 mM NaPO_4_, 0.05% (v/v) Tween20, pH 6.0 or 100 mM NaPO_4_, 0.05% (v/v) Tween20, pH 7.4 three times with 30 minute incubations in between washes. Residual binding of antibodies was detected by a goat Anti-Human IgG (Fab specific) antibody at 1 in 5,000 (Sigma) and were visualized with tetramethylbenzidine (TMB) at OD450nm.

### Pseudovirus Production

Pseudovirus plasmids expressing the HIV Env of interest were co-transfected with pSG3DEnv backbone-expressing plasmids (obtained from the NIH AIDS Research and Reference Reagent Program, Division of AIDS, NIAID, NIH) into HEK293T cells using PEI-MAX 40,000 (Polysciences). Cultures were incubated for 48 hours at 37°C, then supernatants filtered through 0.45 μm and frozen in DMEM/20% FBS to yield Env-pseudotyped viruses capable of a single round of infection only as previously described (56).

### Neutralization Assay

Neutralization assays were performed in TZM-bl cells as previously described (57). Neutralization was measured as a reduction in RLUs after a single round of pseudovirus infection in the presence of the monoclonal antibody. bNAbs were serially diluted 1:3 and the IC_50_ calculated as the dilution at which the infection was reduced by 50%. All subclass switch variants were run head-to-head on the same plate to limit intra-experimental variation. Eleven viruses from the global panel (50) including 246.F3.C10.2, 25710.2.43, 398.F1.F6.20, 703010217.B6, BJOX002000.03.2, CE1176.A3, CH119.10, CNE55, TRO.11, X1632.S2.B10 and X2278.C2.B6 were tested against all bNAbs. For combinations of IgG1 and IgG3 bNAbs, the Bliss-Hill model was calculated by the tool COMBINABER (http://www.hiv.lanl.gov/content/sequence/COMBINABER/combinaber.html) as described in (58).

### SOSIP Trimer ELISA

Avitagged SOSIP trimers were biotinylated using BirA ligase as described elsewhere (59). Biotinylated trimer was coated on to streptavidin ELISA plates (Thermofisher) at 4 μg/ml in PBS and incubated for 1 hour at room temperature. Following PBS washes, the plates were blocked for 30 minutes in 5% milk/PBS and washed in PBS. Fifty μl of bNAb variants as well as negative controls 447-52D and F105 (starting at 10μg/ml) were incubated for 1 hour at room temperature, followed by PBS washes. Secondary antibody, goat anti-human Fab-HRP (Sigma) was incubated in the plate for 1 hour at room temperature, the plate washed three times with PBS and 100μl TMB added to each well. The reaction was stopped with 1M H_2_SO_4_ and read at 450nm.

### Statistical Analysis

Analysis of all flow cytometry based experiments was done using FlowJo (FlowJo LLC, Ashland, OR). Sequencing to confirm cloning was analyzed with Sequencher 5.4.1. All statistical analysis was performed in GraphPad Prism 6 (GraphPad Software, Inc, La Jolla, CA). All comparisons between groups were done with non-parametric tests including Mann-Whitney U tests (for two unmatched groups) and Wilcoxon matched pairs signed rank test (for two matched groups). All confidence intervals were set to 95%. All correlations reported are non-parametric Spearman’s correlations and all statistical analysis was done with two-sided testing with using an alpha level of 0.05.

## Results

### IgG3 Improves Antibody-Dependent Cellular Phagocytosis (ADCP)

**Figure 1:**
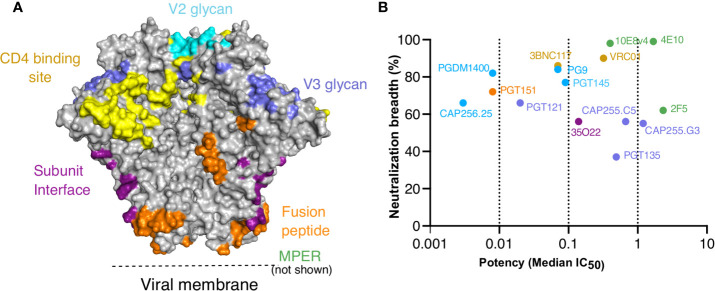
Targets, neutralization breadth and potency of selected HIV broadly neutralizing antibodies. IgG1*01 and IgG3*01 variants of 15 bNAbs that target the six major epitopes of vulnerability were expressed. **(A)** Major contacts of each of the bNAbs on the BG505 SOSIP.664 trimer (PDB: 6V0R). **(B)** Neutralization breadth and potency of IgG1 bNAb variants represented by median IC_50_ (μg/ml) against a 200 multiclade virus panel as obtained from CATNAP.

We and others have previously shown both CAP256-VRC26.25 (referred to as CAP256.25) (34) and VRC01 (45) had improved ADCP when engineered as IgG3. Here we investigated whether this was applicable to bNAbs that targeted other epitopes on the trimer. To do this, we selected 13 additional bNAbs that target different epitopes on the HIV trimer as shown in **Figure 1A**, and included both CAP256.25 and VRC01 as positive controls. These bNAbs, several of which are under clinical development (60), have a wide range of neutralization potencies and breadth as measured against a multiclade 200-virus panel (**Figure 1B**). We cloned the variable regions of each bNAb into IgG1*01 and IgG3*01 antibody heavy chain expression plasmids and expressed both variants head-to-head in HEK293F cells. Following purification, we determined the protein concentration of each antibody, accounting for the different sizes of IgG1 and IgG3.

**Figure 2:**
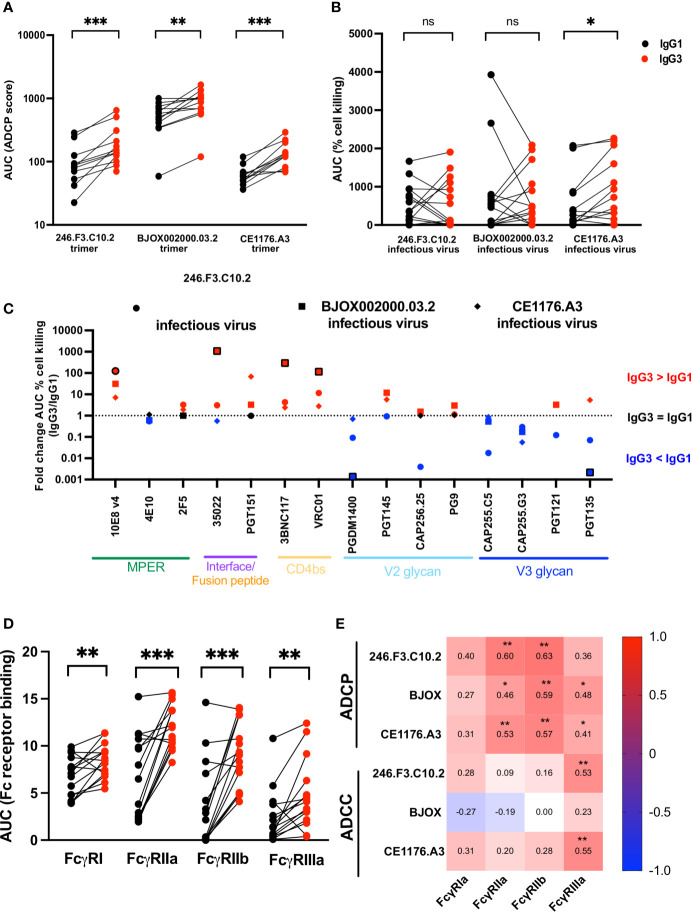
IgG3 bNAb variants show improved ADCP, epitope specific ADCC and Fc receptor binding enhancement compared to IgG1. IgG1 and IgG3 variants of bNAbs were tested for **(A)** antibody-dependent cellular phagocytosis (ADCP) measured against SOSIP trimer coated beads (246.F3.C10.2, BJOX002000.03.2 and CE1176.A3). ADCP scores are represented as the area under the curve (AUC) and MPER-specific bNAbs are not included. **(B)** Antibody-dependent cellular cytotoxicity (ADCC) measured as the cell killing of CEM.NKR.CCR5 cells infected with Renilla-expressing infectious molecular clones 246.F3.C10.2, BJOX002000.03.2 and CE1176.A3. ADCC activity represented here as the area under the curve of the percentage reduction of luminescence. Red indicates IgG3 and black IgG1. **(C)** Fold change of the AUC of ADCC cell killing between IgG3 and IgG1 bNAbs targeting multiple epitopes, with the shapes indicating the virus used to infect target cells. Red indicates instances where IgG3 was more effective at mediating ADCC, with blue indicating IgG1 showed greater ADCC than IgG3, and black those cases where the difference equals 1 fold. Outlined shapes indicate instances where there is a knock-out of activity of one IgG variant. **(D)** Fc receptor binding of IgG1 (black) and IgG3 (red) bNAb variants measured by ELISA and represented as AUC and **(E)** Spearman’s correlations of antigen-specific ADCP and ADCC and Fc receptor binding of IgG1 and IgG3 variants. Wilcoxon matched pairs signed rank t test were used to compare IgG1 and IgG3 activity with significance indicated as *p < 0,05; **p < 0,01; ***p < 0,001; ns, non-significant. Plots are representative of at least two independent experiments.

All 15 bNAb pairs were tested for ADCP activity against three SOSIP trimers from the global panel; 246.F3.C10.2 (clade AC), BJOX002000.03.2 (clade CRF07) and CE1176.A3 (clade C) (**Figure 2A** and **Supplementary Figures 1A–F**). We compared two scores of ADCP activity, area under the curve (AUC) which encompasses the full titration, or activity measured at 10 μg/ml, the highest concentration tested. Both measures of ADCP activity were highly correlated for all three trimers (**Supplementary Figures 1B, D, F**), and therefore we used AUC in all subsequent analyses. IgG3 bNAb variants showed significantly improved ADCP compared to IgG1, for all three trimers (**Figure 2A**). In line with previous studies (34, 45), though against different antigens, we confirmed higher ADCP for both VRC01 and CAP256.25 IgG3 variants compared to IgG1 (**Supplementary Figures 1A, C, E**). As SOSIP trimers lack the membrane-proximal external region (MPER), the MPER bNAbs showed no ADCP activity against the trimers (**Supplementary Figures 1A, C, E**). In order to test their ADCP activity, MPER bNAbs were tested using an MPER consensus peptide MPR.03. Unlike the other bNAbs, MPER bNAbs overall showed no significant difference in IgG3 compared to IgG1, perhaps because MPR.03 is linear, rather than being in a native structural conformation (**Supplementary Figures 1G, H**).

In order to investigate whether the higher IgG3 ADCP activity could be attributed to differences in binding to the trimer, we measured their ability to bind 246.F3.C10.2, BJOX002000.03.2 and CE1176.A3 trimers by ELISA (**Supplementary Figure 2**). bNAb pairs that failed to bind the trimer, or did so weakly, were unable to mediate high levels of ADCP. Examples include PGT135 against 246.F3.C10.2 (**Supplementary Figures 1A** and **2A**), 3BNC117 against BJOX002000.03.2 (**Supplementary Figure 1C** and **2B**) or PGT145 against CE1176.A3 (**Supplementary Figures 1E** and **2C**). The IgG1 and IgG3 MPER bNAbs showed no difference in binding to the MPR.03 peptide (**Supplementary Figure 2D**), reflecting their lack of difference in ADCP against this antigen. While binding was significantly correlated with ADCP mediated by IgG1 bNAbs these were not observed for IgG3 (**Supplementary Figure 2E**). We also observed no significant change in trimer binding between IgG1 and IgG3 variants (**Supplementary Figure 2F**). Together this data shows that enhanced ADCP activity of IgG3 bNAbs is not simply a result of improved binding to trimer but likely an enhancement in avidity, perhaps through the longer hinge as shown elsewhere (34, 45).

### IgG3 Mediated Antibody-Dependent Cellular Cytotoxicity (ADCC) Is Enhanced in an Epitope-Specific Manner

We next assessed the effect of IgG3 on the ability of bNAbs to mediate ADCC. We used infectious Renilla reporter viruses made from 246.F3.C10.2, BJOX002000.03.2 and CE1176.A3 in the Env-IMC-6ATRi backbone. These were used to infect a lymphocytic cell line CEM.NKR.CCR5 which showed substantial downregulation of CD4 and >30% infection of the cells as determined through p24 expression (**Supplementary Figure 3A**) compared to a mock infection. ADCC was measured as the decrease in luminescence of cells in the presence of antibody and effector PBMCs, relative to a “no mAb” control, representative of the killing of infected target cells.

In contrast to ADCP, ADCC of IgG3 variants did not show overall improvement compared to IgG1 (**Figure 2B**, **Supplementary Figures 3B–G**). As for ADCP, area under the curve was used to represent ADCC which also correlated with peak ADCC activity (**Supplementary Figures 3C, E, G**). However, while ADCC activity against CE1176.A3 was significantly improved by IgG3, particularly for PGT151, 10E8v4, 3BNC117 and VRC01 (**Supplementary Figure 3F**), this was not true of 246.F3.C10.2 or BJOX002000.03.2, where mixed isotype-driven effects were observed.

To understand epitope effects, we assessed the effect of IgG3 variant for each bNAb pair, represented as fold change between IgG3 and IgG1 (**Figure 2C**). Interface and CD4 binding site directed bNAbs, particularly, showed an overall improvement of ADCC when expressed as IgG3. This was especially true for 3BNC117 and VRC01, where all three viruses tested showed improved ADCC as IgG3. MPER bNAbs 10E8v4 and 2F5 also showed improved ADCC as IgG3 with the former showing significant improvement. In contrast, PGDM1400 and both CAP255 mAbs showed increased ADCC as IgG1 variants. This finding suggests that engineering bNAbs as IgG3 to improve ADCC is both virus- and bNAb-specific.

### Fcγ Receptor Binding Is Enhanced by IgG3

We next examined the affinity of the IgG1 and IgG3 pairs for Fc receptors, as this modulates Fc effector function, and IgG3 mAb variants have increased binding to Fcγ receptors compared to IgG1 (61). One of the major contributors to differences in Fc receptor binding is Fc glycosylation which differs by cell line in which the antibody is produced (62). We therefore tested IgG1 and IgG3 variants produced in the same cell line, head to head. We tested bNAb binding to FcγRI, FcγRIIa, FcγRIIb and FcγRIIIa by ELISA, and showed that IgG3 showed significantly improved binding to all receptors tested (**Figure 2D** and **Supplementary Figures 4A–D**). However, Fc receptor binding levels varied for different bNAbs despite the fact that all utilize the same IgG1 or IgG3 backbones, suggesting a role for Fab-Fc interactions in determining Fc receptor affinity (**Supplementary Figure 4**).

We next assessed the relationship between binding to Fc receptors and functional assays as it is known that FcγRIIa and FcγRIIb modulate ADCP function (63), whereas FcγRIIIa mediates ADCC (64). In line with this, bNAb binding to FcγRIIa and FcγRIIb correlated with ADCP activity across all SOSIP trimers tested and FcγRIIIa binding correlated with ADCC activity (**Figure 2E**). However, Spearman’s correlations were relatively low (less than or equal to 0.6) perhaps suggesting that factors other than Fc receptor binding contribute to the enhanced activity of IgG3 version of these antibodies.

### pH Dependence for Binding to the Neonatal Fc Receptor Is Similar for IgG1 and IgG3 bNAbs That Use a Lambda Light Chain

One of the major reasons that IgG3 is not widely considered for therapeutic use is that it has a reduced half-life of approximately 7 days compared to that of 21 days for IgG1 (41). It is known that the reduced pH dependence of IgG3 compared to IgG1 is the major contributor to poor half-life, with antibody binding to FcRn being a good proxy of half-life (39).

**Figure 3:**
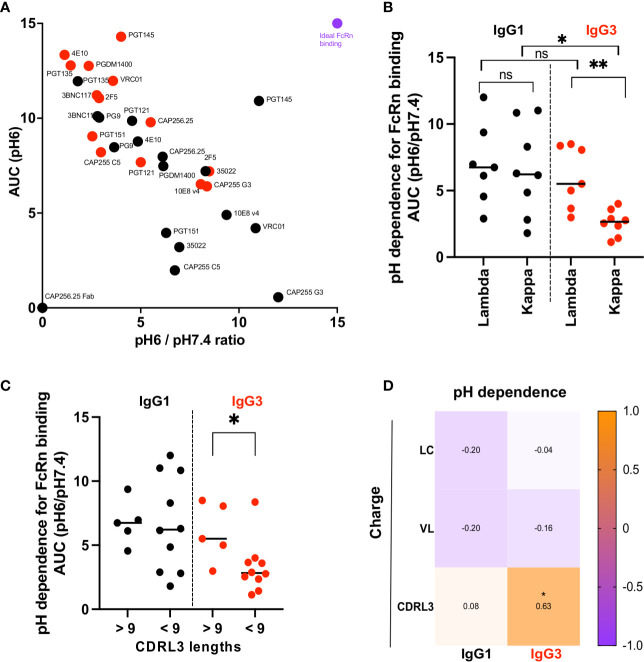
IgG3 bNAb variants with a Lambda light chain show significantly improved dependence on pH for FcRn binding. **(A)** Binding to the FcRn receptor measured by ELISA at pH6 and pH7.4. Y axis shows binding to the receptor at pH6 area under the curve (AUC) and the X axis the ratio of the area under the curve of binding to the FcRn receptor at pH6 and pH7.4. Red dots indicate the IgG3 and black the IgG1 bNAb variants. Purple denotes an example of an antibody with optimal FcRn binding. **(B)** pH dependence (AUC pH6/pH7.4) for antibodies with lambda or kappa light chains. Significance is indicated by a Mann Whitney t test for unpaired samples (** denotes p < 0,01) and a Wilcoxon paired test for paired samples (* denotes p < 0,05). **(C)** pH dependence (AUC pH6/pH7.4) for antibodies with CDRL3 lengths below and above 9 are shown with significance indicated by a Mann Whitney t test where * denotes p < 0,05. **(D)** Net charge at pH7.4 of the light chain (LC), the variable region of the light chain (VL) and the complementarity determining region 3 of the light chain (CDRL3) correlated with the pH dependence of IgG1 and IgG3 bNAbs (represented as AUC pH6/pH7.4) are shown as Spearman’s correlations where * denotes p < 0.05 and ns denotes non-significant.

We measured the relative abilities of IgG3 and IgG1 bNAbs to bind FcRn by ELISA at pH 6 and pH 7.4, to establish pH dependence. IgG3 bNAbs generally showed increased binding to FcRn under acidic conditions (measured as area under the curve at pH 6) but reduced pH dependence, defined as the ratio of binding at pH 6 and pH 7.4 (**Figure 3A** and **Supplementary Figure 5A**).

The observation that bNAbs with shared constant regions showed differential pH dependence suggests that the variable region impacts antibody binding to the FcRn receptor. Several studies have shown that the light chain affects FcRn binding (48, 49). Comparison of the bNAbs showed that although IgG1 bNAbs were similar in their pH dependence for FcRn binding regardless of whether they used kappa or lambda light chains, there was a striking difference in pH dependence of IgG3 bNAbs based on light chain use (**Figure 3B**). Specifically IgG3 bNAbs using kappa light chains were significantly less pH dependent for FcRn binding than those with a lambda light chains. We further show that IgG3 bNAbs exhibiting enhanced pH dependence were enriched for CDRL3s greater than 9 amino acids in length (**Figure 3C**) and that lambda light chains were significantly longer overall (**Supplementary Figure 5B**). Finally we examined whether the charge of the light chain, or different regions thereof, impacted the pH dependence of IgG3 bNAbs. While there was no correlation between the net charge at pH7.4 of the entire light chain or of the variable region of the light chain for either IgG1 or IgG3, the charge of the CDRL3 for IgG3 bNAbs was significantly correlated with improved pH dependence (**Figure 3D**).

Overall, this indicates that CDRL3 charge and length is associated with increased pH dependence in IgG3 bNAbs, which results in binding profiles similar to that of IgG1.

### IgG3 Enhances or Maintains Neutralization Potency of bNAbs

Although Fc receptor binding contributes to protection afforded by bNAbs (65), the major mechanism for protection is neutralization. We therefore measured neutralization of IgG1 and IgG3 bNAb pairs against 11 viruses from the multiclade global virus panel, widely used to define broadly neutralizing sera in HIV infected individuals (50).

**Figure 4:**
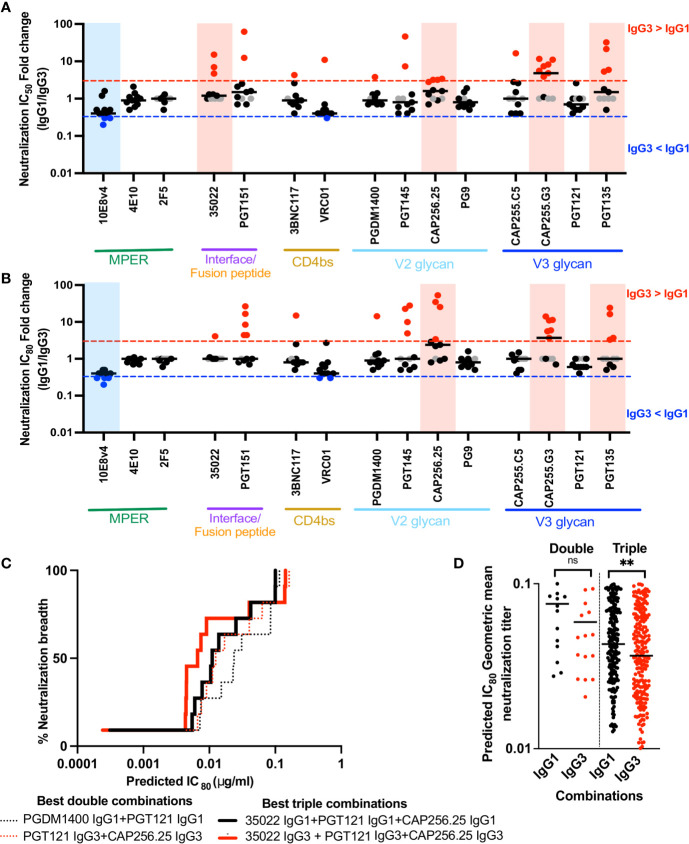
IgG3 bNAb variants enhance or maintain neutralization of a multiclade panel of viruses and IgG3 combinations are are more potent that IgG1 combinations. **(A)** Fold changes of IC_50_ and **(B)** IC_80_ between IgG3 and IgG1 bNAb variants, by epitope targets against 11 viruses from the multiclade global panel where the neutralization is represented by individual dots. The red and blue lines indicate 3 fold difference where IgG3 > IgG1 and IgG3 < IgG1 respectively. Grey dots indicate instances where a virus is resistance to neutralization and shaded blue and red areas indicating those instances where there is a statistically significant difference between IgG1 and IgG3 activity against all viruses, for that bNAb pair. **(C)** Neutralization breadth potency curves of the best double (dotted) and triple (bold) combinations of antibodies as defined by the Bliss-Hill model. IgG1 in black, IgG3 in red. **(D)** All double or triple combinations of antibodies that show 100% breadth against the 11 virus panel are shown as the predicted IC_80_ geomean titers. ** denotes p < 0.01 and ns denotes non-significant, Mann-Whitney t test.

Overall, these bNAbs either maintained neutralization activity (1-3 fold differences in either direction) or showed improved neutralization (>3 fold enhancement compared to IgG1) across several viruses at both IC_50_ and IC_80_ (**Figures 4A, B** and **Supplementary Figure 6A**). BNAbs 35O22, CAP255.G3, PGT135 and, as we previously showed CAP256.25 (34) showed significant improvement across the 11 viruses tested as IgG3 at IC_50_ (**Figure 4A** and **Supplementary Figure 6B**) with 35O22 losing significance at IC_80_ owing to its plateau (**Figure 4B**). An exception was 10E8v4, which despite the fact that the parent antibody, 10E8, was originally isolated as an IgG3 (66), showed reduced neutralization as IgG3 at both IC_50_ and IC_80_.

Binding of IgG1 bNAbs to the global panel trimers 246.F3.C10.2, BJOX002000.03.2 and CE1176.A3 generally reflected the neutralization of these viruses as expected (**Supplementary Figures 2C–E**). BNAbs unable to neutralize were similarly unable to bind the trimer at all or bound at low levels, with the exception of 35O22 which bound strongly to the BJOX002000.03.2 SOSIP trimer but was unable to neutralize it. This may be as a result of differences in the conformation of the soluble SOSIP trimer compared to the trimer on viral particles. However, despite differences in neutralization between IgG1 and IgG3, their capacity to bind was generally similar for the three viruses we tested (**Supplementary Figure 2F**). This may be because neutralization is impacted by factors in addition to trimer binding, such as the potential ability of IgG3 bNAbs which have increased rotational freedom, to cross-link trimers on a virus, compared to IgG1.

### IgG3 bNAbs Show More Potent Neutralization in Combination Than IgG1

Interventions based on passive immunization of bNAbs will likely require combinations of antibodies that target different epitopes in order to overcome viral resistance (58). We thus investigated whether the increase in neutralization potency of some IgG3 bNAbs conferred a benefit in combinations of bNAbs. We used the Bliss-Hill model (58) to predict the best double and triple combinations of either IgG1 or IgG3 bNAbs against the global virus panel. While the best triple combination of either IgG1 or IgG3 included the same bNAbs (35O22+PGT121+CAP256.25), the best double combination for IgG1 bNAbs was PGDM1400+PGT121 whereas for IgG3, it was PGT121+CAP256.25 (**Figure 4C**). The best IgG3 double and triple combinations were more potent than the best IgG1 combinations. Furthermore, when all combinations that showed 100% breadth were compared, IgG3 bNAbs showed a trend to increased potency in double combinations, and significantly improved potency in triple combinations (**Figure 4D**). Therefore, the potency of combinations may be further enhanced through as the use of IgG3 bNAbs, to improve both neutralization and Fc effector function.

## Discussion

The contribution of the constant region of bNAbs has emerged as being important for optimal protection from infection as well as for the elimination of already infected cells (67, 68). Given our previous observation that both the Fc effector function and the neutralization potency of CAP256.25, a V2-directed bNAb, was significantly enhanced as an IgG3 variant (34), we expanded this to additional bNAbs. We show that the IgG3 isotype enhances ADCP and, in an epitope specific manner, ADCC, for several bNAbs. Furthermore, IgG3 maintains or improves neutralization against a small but globally relevant panel. This suggests the potential broader applicability of this subclass in passive immunization.

Fc receptor mediated cytotoxic functions are likely to be beneficial at transmission, especially at the mucosa where phagocytes predominate (69). In line with several previous studies (34, 43, 45), we show that IgG3 bNAb variants show enhanced ADCP compared to matched IgG1 variants. We further show that this enhancement could not be attributed to increased binding of IgG3 variants to trimer, similar to what has been observed for other bNAbs (34, 43, 45). We also found that IgG3 binding to FcγRIIa, the Fc receptor that largely facilitates ADCP, was significantly increased compared to IgG1. This is in contrast to a previous study that saw no differences in IgG1 or IgG3 bNAb binding to Fc receptors or antigen by surface plasmon resonance (45). These discrepancies may be a consequence of the use of ELISA, and it is also possible that variation in Fc glycosylations as a result of cell line used may result in differences in binding. We note that in our study, although there is a correlation between FcγRIIa binding and function, this was not very strong, suggesting other features of the antibody may contribute to increased ADCP. One possibility is that the longer IgG3 hinge may result in cross-linking of antigen. Ultimately, while improved ADCP as IgG3 has previously been studied in a small number of HIV bNAbs (34, 43, 45), we show here that it is broadly applicable across bNAbs targeting different epitopes.

While IgG3 showed marked improvement of ADCP regardless of the antigen that was tested, or the epitope targeted, ADCC capacities of IgG3 bNAb variants were epitope and antigen specific. Overall, while ADCC activity was not uniformly enhanced by IgG3, the isotype was advantageous for several MPER, gp41-gp120 interface and CD4 binding site bNAbs. This is in line with previous studies that show highly virus specific IgG1 bNAb ADCC activity which was dependent on the ability of these antibodies to bind infected cells (67, 70–72). In a study that examined the ADCC of bNAbs, those that induced the strongest FcγRIIIa stimulation were most active (67), which is similar to our study where ADCC correlated with binding to this Fc receptor. Given that antibody angle-of-approach influences how the Fc is presented to the receptor and the level of avidity induced (73, 74), the increased reach of IgG3 may be advantageous against certain epitopes. Similarly the nature of the distribution of trimer on the surface of the cell, which likely differs between viruses, may influence Fc presentation as it does for other pathogens (75). This may explain the virus dependent ADCC enhancement seen between IgG1 and IgG3. While the hinge length of IgG3 does not influence the ADCC of VRC01 (45), it is interesting that short hinge variants of non-HIV antibodies show enhanced ADCC, confirming that this region should be further investigated in HIV bNAbs (38). Further, beyond ADCP and ADCC other Fc effector functions should be investigated. Given IgG3 can enhance complement-mediated lysis relative to IgG1, specifically in cases of sparse antigen density (76), and this function is important for HIV bNAb mediated killing (70), it is likely of relevance.

The reduced half-life of IgG3, compared to IgG1, has limited their potential as therapeutics. This shorter half-life is determined by its low pH dependence during FcRn-mediated recycling (39). In this study we show that as expected, IgG3 bNAbs overall display lower pH dependence compared to IgG1. However, individual bNAbs showed significantly different binding profiles, indicating the involvement of the Fab in FcRn binding. Several previous studies have shown this, either by comparing antibodies with identical Fc portions and different variable regions (77, 78) or using the hydrogen deuterium exchange method (79). Minor contributions from complementarity determining regions light chain 3 (CDRL3) of antibodies have been shown to strengthen the interaction with the FcRn (48, 49). Particularly for IgG3 it is possible that residues in CDRs could directly contact FcRn following the first interaction at the putative binding site, owing to the inherent flexibility of the Fab arms (41). Here we show that IgG3 dependence on pH was significantly lower for those bNAbs that use a kappa light chain, in contrast to those that use a lambda light chain. Given that IgG1 bNAbs showed no such difference, this preference appears to be unique to the IgG3 molecule. There was no difference in pH dependence between lambda IgG3 bNAbs and matched IgG1. This suggests these may have similar half-lives to IgG1 *in vivo*, although this yet to be assessed. Lambda light chains overall showed longer CDRL3 chains, which along with charge was associated with improved pH dependence of IgG3 bNAbs. These data suggest that the Fab portion may strengthen FcRn binding and compensate for the otherwise poor pH dependence of IgG3 constant regions.

We further showed that 14 of the 15 bNAb pairs tested in this study, to multiple epitopes, showed either enhancement or maintenance of neutralization activity as IgG3, with only 10E8v4 showing loss of neutralization. This extends previous studies showing that the constant region can alter neutralization potency of bNAbs (30, 32–34, 80, 81). As in previous studies VRC01 as well as MPER bNAbs 2F5 and 4E10 which were isolated as IgG3 (45, 82, 83), showed no difference in potency between IgG1 and IgG3. While for several bNAbs, we find large fold differences in neutralization potency between IgG1 and IgG3, binding to a subset of three trimers showed minimal difference. This suggests other mechanisms for enhanced neutralization, which may include cross linking of trimers or intra-trimeric binding, with the longer hinge length of IgG3 likely able to increase Fab–Fc distance, Fab–Fab distance and flexibility (40). A key immune evasion mechanism of HIV-1 against host antibody responses is the remarkably low density of Env molecules on the viral surface (84), a feature that IgG3 is able to circumvent in other pathogens due to its enhanced reach (75). This is supported in HIV by data showing that bispecific bNAbs, particularly 3BNC117/PGT135, with engineered hinge lengths equivalent to that of IgG3, showed increased neutralization potency by favoring intra-trimeric, bivalent interactions and increasing avidity of the construct (85). Interestingly, the IgG3 variant of PGT135 also showed significant enhancement in our study. Overall our findings suggest that the unique structure of IgG3 may be an advantage for neutralization.

For passive immunization strategies, there is an increased focus on combinations of antibodies to increase viral coverage and counteract resistance. We show that combinations of IgG3 bNAbs show enhanced potency, compared to IgG1 combinations against the small panel of viruses tested. In addition to enhanced neutralization, combinations of potent IgG3 bNAbs would also have the advantage of increased Fc effector function, which may be advantageous for future passive immunization strategies. This represents an alternative strategy to the engineering of specific residues within IgG1 bNAbs to increase effector function, an approach which has shown variable improvement in protection *in vivo* (25).

The paucity of therapeutic IgG3 bnAbs is largely borne out of concerns about half-life, which we show here also depends on the Fab portion of the antibody. These findings may enable strategies to mitigate the poor pH dependence of IgG3. An additional concern frequently raised is allotypic mismatch resulting in adverse reactions owing to the polymorphic nature of IgG3. However as many of the changes are isoallotypic (occurring in other IgG subclasses) (29) this is unlikely to be a major issue in passive immunization. The stability of IgG3 can also be improved through mutations in the CH3 (86). Most importantly, IgG3 has shown tolerability and benefit in humans for the treatment of lung cancer and *Staphylococcus aureus* bacteraemia (41). Our study shows that the polyfunctional enhancement of bNAbs by IgG3 is substantial and these should be considered for passive immunization. Ultimately, this study leverages the IgG3 subclass to improve antibody function and shows that antibodies should be optimised based not only on their antigen binding characteristics but also on the intrinsic properties of their constant regions.

## Data Availability Statement

The original contributions presented in the study are included in the article/**Supplementary Material**. Further inquiries can be directed to the corresponding author.

## Ethics Statement

The studies involving human participants were reviewed and approved by Human Research Ethics Committee of the University of the Witwatersrand. The patients/participants provided their written informed consent to participate in this study.

## Author Contributions

SR conceptualized, performed experiments, analyzed data, generated the figures and wrote the manuscript. FA, BO, ZM, and BL produced antibodies and proteins. NM performed Fc experiments. LM and PM assisted in data interpretation. PM wrote the manuscript. All authors contributed to the article and approved the submitted version.

## Funding

SR and PM are supported by the South African Research Chairs Initiative of the Department of Science and Innovation and National Research Foundation of South Africa, the SA Medical Research Council SHIP program, the Centre for the AIDS Program of Research (CAPRISA) and an H3 Africa grant (U01A136677). SR is also supported by the Poliomyelitis Research Foundation and is a L’Oreal/UNESCO Women in Science South Africa Young Talents awardee. The funder was not involved in the study design, collection, analysis, interpretation of data, the writing of this article or the decision to submit it for publication. Related research by the authors is conducted as part of the DST-NRF Centre of Excellence in HIV Prevention, which is supported by the Department of Science and Technology and the National Research Foundation.

## Conflict of Interest

The authors declare that the research was conducted in the absence of any commercial or financial relationships that could be construed as a potential conflict of interest.

## Publisher’s Note

All claims expressed in this article are solely those of the authors and do not necessarily represent those of their affiliated organizations, or those of the publisher, the editors and the reviewers. Any product that may be evaluated in this article, or claim that may be made by its manufacturer, is not guaranteed or endorsed by the publisher.
