# Supplementary material for: HIV Broadly Neutralizing Antibodies Expressed as IgG3 Preserve Neutralization Potency and Show Improved Fc Effector Function
Source: Front Immunol. 2021 Sep 10;12:733958. doi: 10.3389/fimmu.2021.733958 (PMC8462932; doi:10.3389/fimmu.2021.733958)
Supplement: Supplementary file 1 [file DataSheet_1.docx]

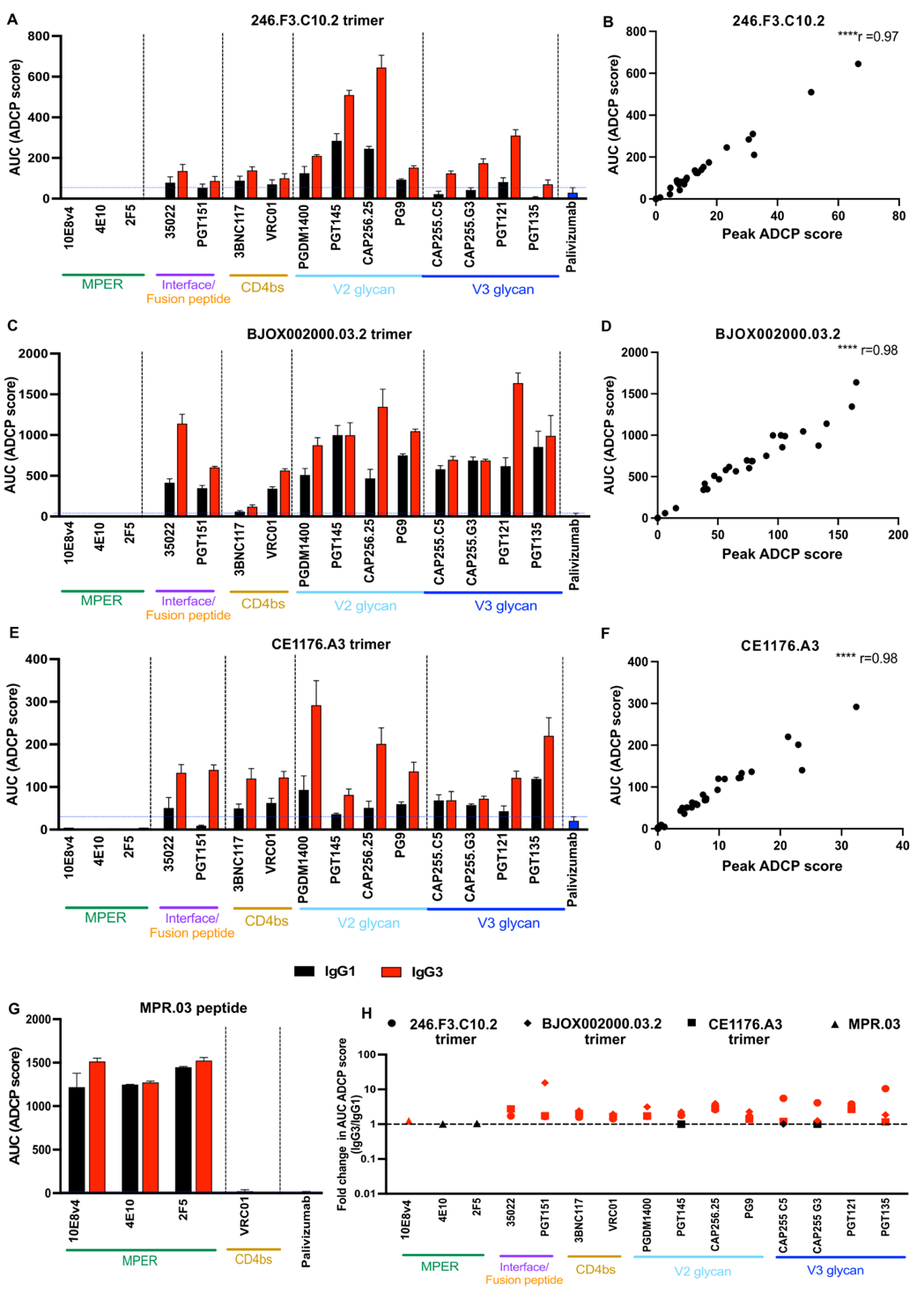


**Supplementary Figure 1: Antibody dependent cellular phagocytosis of IgG1 and IgG3 bNAb variants**

ADCP is represented as area under the curve for five-fold titrations from 10-0,08μg/ml for all antibodies tested against SOSIP trimers derived from the global panel viruses 246.F3.C10.2, BJOX002000.03.2 and CE1176.A3 (**A,C,E**). Endpoint values at the highest concentration of bNAb tested (10μg/ml) were significantly correlated with AUC for each antigen (**B,D,F**). (**G**) ADCP for the MPER bNAbs are shown against the MPR.03 peptide with VRC01 and Palivizumab as the negative controls. Bars represent the mean and the error bars indicate the standard deviation of a minimum of two independent experiments. Red bars indicate IgG3 and black bars indicate IgG1. (**H**) Fold change of the AUC of ADCP between IgG3 and IgG1 bNAbs targeting diverse epitopes are shown, with the shapes indicating the relevant SOSIP trimer, red indicating those instances where IgG3 was more effective at mediating ADCC, with black indicating those cases where the difference is equal to 1 fold.


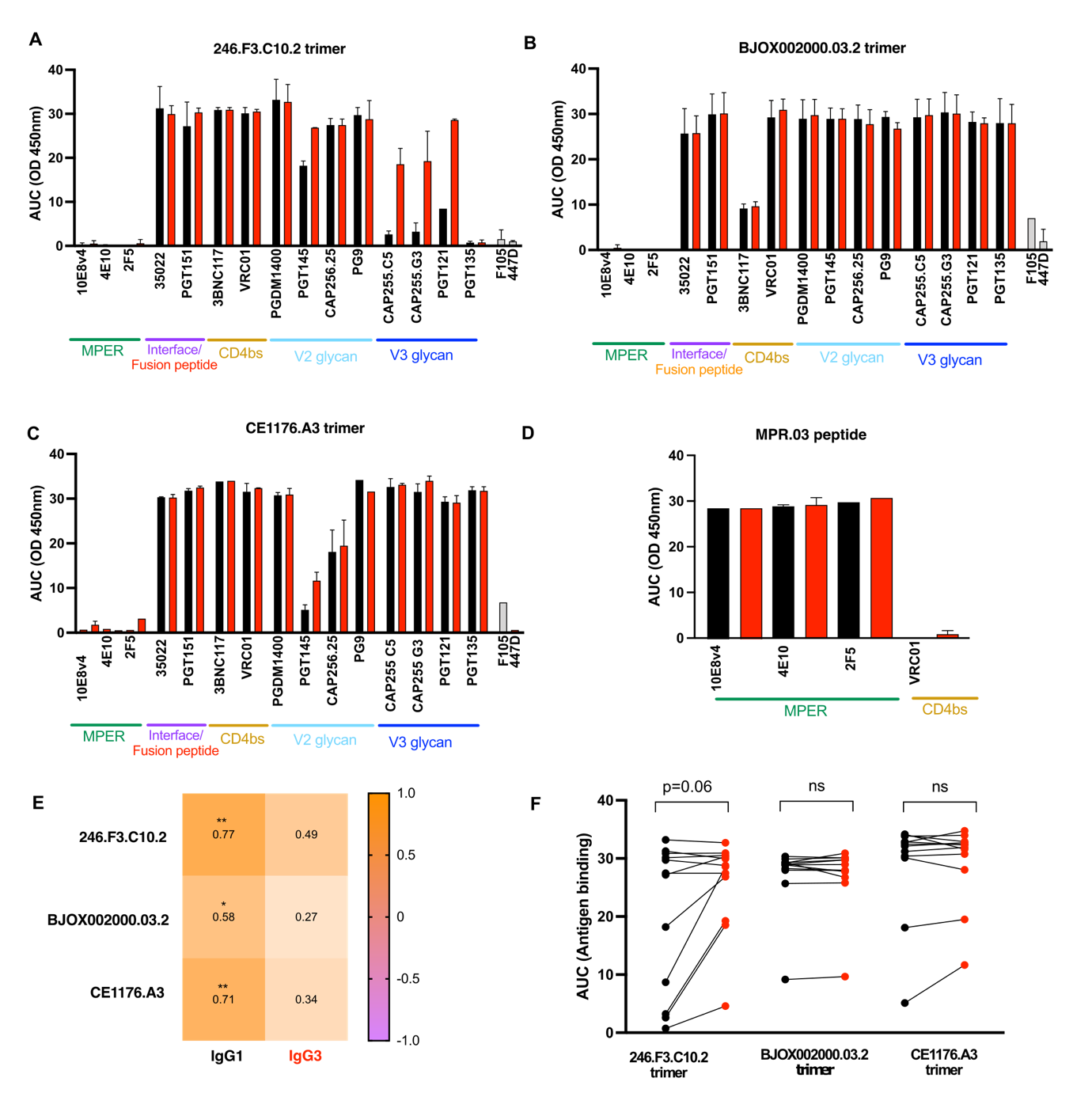


**Supplementary Figure 2: Antigen binding of IgG1 and IgG3 variants by ELISA**

Binding of IgG1 (black) and IgG3 (red) bNAb variants by ELISA to SOSIP trimers from global panel viruses (**A**) 246.F3.C10.2, (**B**) BJOX002000.03.2 and (**C**) CE1176.A3 as represented by area under the curve of 6 five-fold titrations from 10μg/ml. Monomeric binding antibodies F105 and 447D (grey) were used as negative controls in the ELISA and represent the mean of two independent experiments. (**D**) Binding by ELISA of IgG1 and IgG3 MPER bNAbs to MPR.03 peptide as represented by area under the curve of 6 five-fold titrations from 10μg/ml. VRC01 was used as a negative control. (**E**) A heatmap of Spearman’s correlations between SOSIP trimer-specific ADCP (AUC ADCP score) and trimer binding (AUC OD450nm) by IgG1 and IgG3 antibodies where *p<0.05 and **p<0.01. (**F**) Binding as represented overall for each trimer (significance calculated as per Wilcoxon signed rank t test).


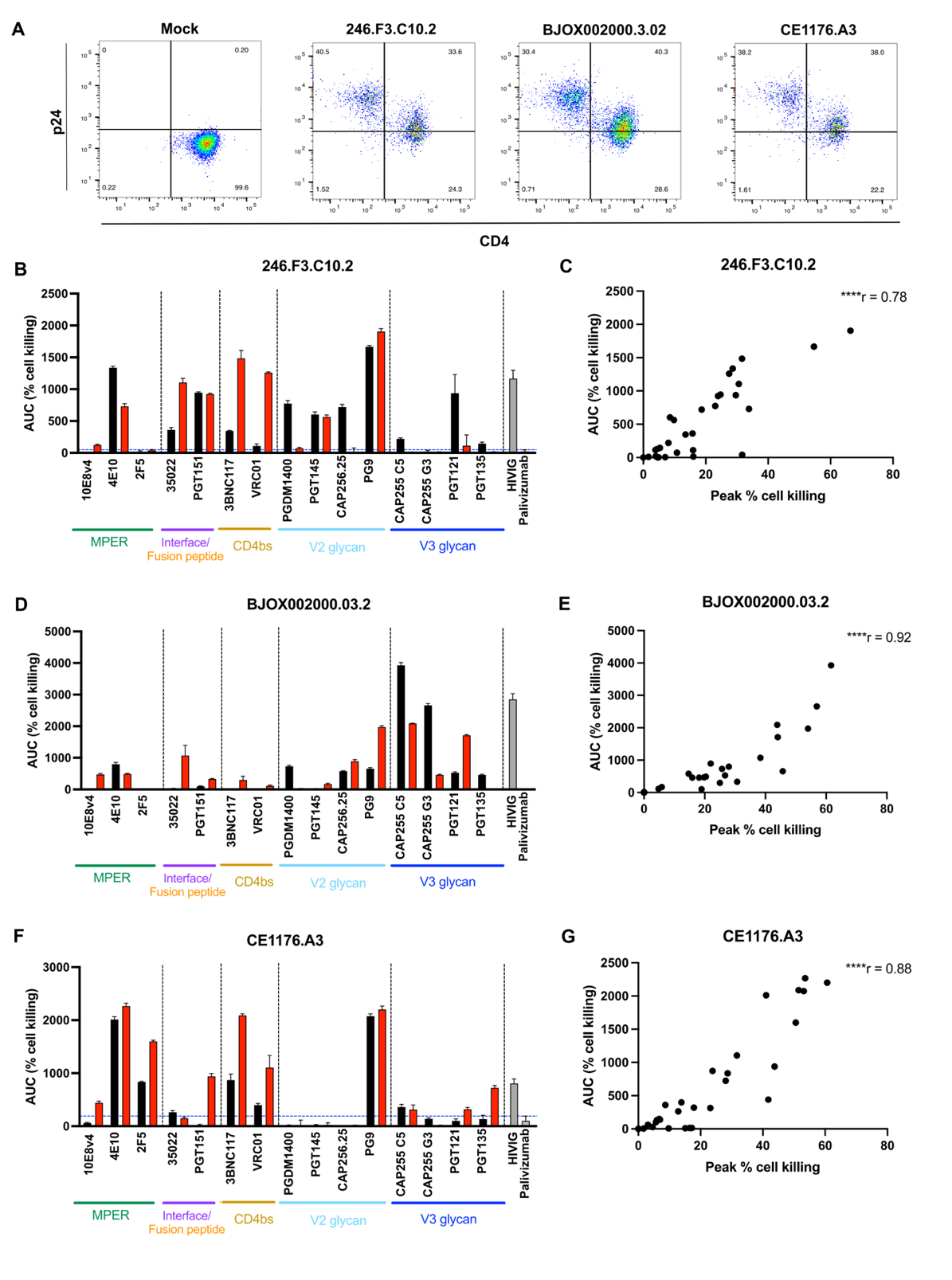


**Supplementary Figure 3:** **Antibody dependent cellular cytotoxicity of IgG1 and IgG3 bNAb variants**

(**A**) p24 levels and CD4 down regulation is shown for CEM.NKR.CCR5 cells infected with 246.F3.C10.2, BJOX002000.03.2 and CE1176.A3 viruses in the NL4-LucR.6ATRi.ecto backbone. (**B,D,F**) ADCC is represented as area under the curve for fivefold titrations from 50-0,04μg/ml for all antibodies tested against CEMS.NKR.CCR5 cells infected with global panel viruses 246.F3.C10.2, BJOX002000.03.2 and CE1176.A3. Bars represent the mean and the error bars indicate the standard deviation of a minimum of two independent experiments where red bars indicate IgG3 and black bars indicate IgG1. (**C,E,G**) Peak values were significantly correlated with AUC against each virus.


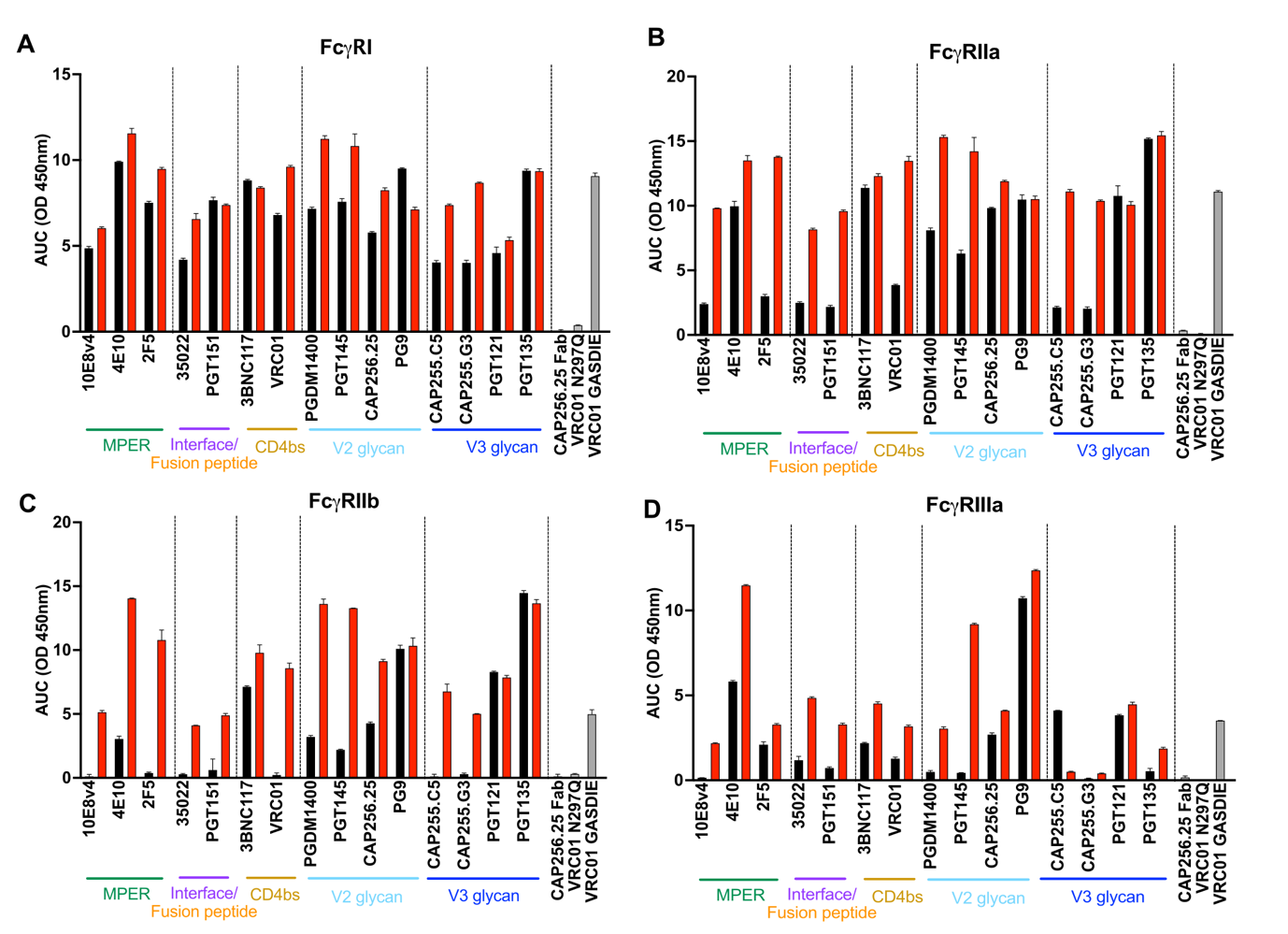


**Supplementary Figure 4: Fc receptor binding of IgG1 and IgG3 bNAb variants**

Fc receptor binding by ELISA was measured for each antibody to (**A**) FcγRI, (**B**) FcγRIIa, (**C**) FcγRIIb and (**D**) FcγRIIIa. Binding is represented as area under the curve of fivefold titrations from 5μg/ml. Negative controls included the Fab portion of CAP256.25 and VRC01 N297Q. The positive control was VRC01 GASDIE an Fc mutated version of VRC01 known to improve Fc receptor binding (S239D/I332E/G236A). Bars are representative of the mean of at least 2 independent experiments.


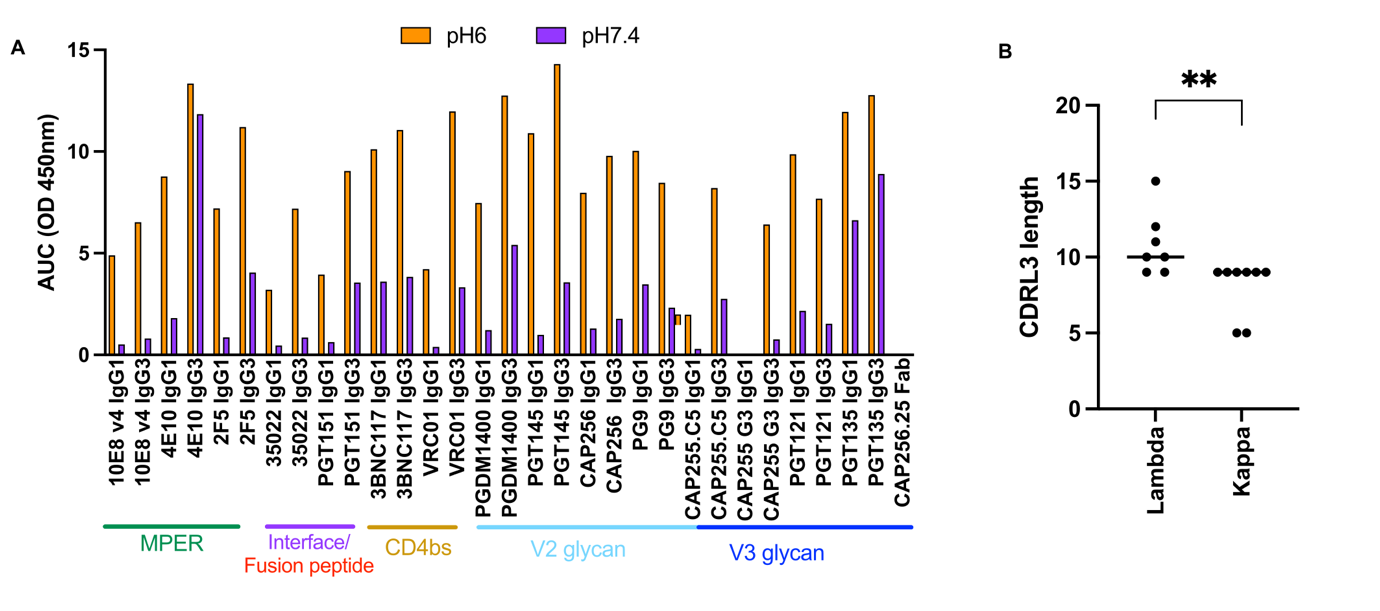


**Supplementary Figure 5: ELISA binding of IgG1 and IgG3 bNAb variants to the FcRn receptor**

(**A**) Binding as measured by ELISA to the FcRn receptor is shown as area under the curve of 5 fold dilutions from 5μg/ml of bNAb variant at pH6 (orange) and pH7.4 (purple). Antibodies are shown separated into their targets on the HIV glycoprotein. (**B**) CDRL3 lengths of kappa and lambda bNAbs where **p<0.01, Mann Whitney t test.


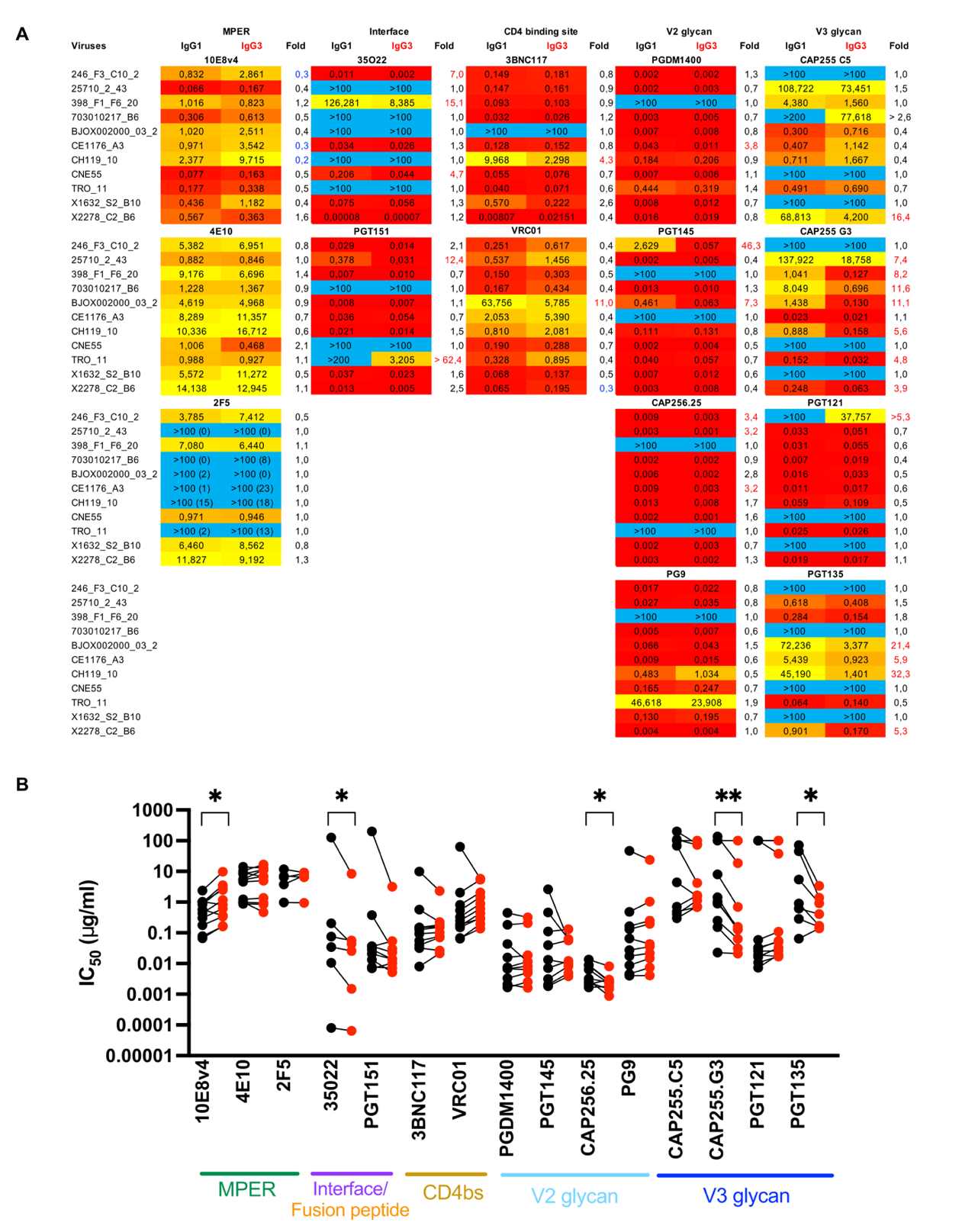


**Supplementary Figure 6: Neutralization of IgG1 and IgG3 bNAb variants**

(**A**) IC_50_ neutralization titers against an 11 virus multiclade panel with fold differences indicated as IgG1/IgG3 IC50s. All folds greater that 3 fold are highlighted. This data is representative of a minimum of 3 independent experiments. (**B**) IC_50_ neutralization titers of IgG1 (black) and IgG3 (red) bNAb variants for 11 multiclade viruses from the global virus panel for which titres against resistant viruses are removed. Results are representative of 3 independent experiments and significance calculated with Wilcoxon sign-ranked paired t test where *p<0.05 and **p<0.01.
